# Magnetic Compression of Tumor Spheroids Increases Cell Proliferation In Vitro and Cancer Progression In Vivo

**DOI:** 10.3390/cancers14020366

**Published:** 2022-01-12

**Authors:** Gaëtan Mary, Brice Malgras, Jose Efrain Perez, Irène Nagle, Nathalie Luciani, Cynthia Pimpie, Atef Asnacios, Marc Pocard, Myriam Reffay, Claire Wilhelm

**Affiliations:** 1 Laboratoire Matière et Systèmes Complexes (MSC), UMR 7057, CNRS and Université de Paris, 75013 Paris, France; gaetan.mary92@gmail.com (G.M.); Irene.Nagle@univ-paris-diderot.fr (I.N.); nathalie.luciani@univ-paris-diderot.fr (N.L.); atef.asnacios@univ-paris-diderot.fr (A.A.); myriam.reffay@univ-paris-diderot.fr (M.R.); 2 Department of Digestive Surgery, Lariboisière Hospital, Université de Paris, UMR 1275 CAP Paris-Tech, AP-HP, 75010 Paris, France; bricemalgras@gmail.com (B.M.); cynthia.crocheray@inserm.fr (C.P.); marc.pocard@inserm.fr (M.P.); 3 Laboratoire Physico Chimie Curie, Institut Curie, PSL Research University-Sorbonne Université—CNRS, 75005 Paris, France; efra.pero@gmail.com

**Keywords:** cancer spheroids, forces and cancer, magnetic nanoparticles, magnetic force

## Abstract

**Simple Summary:**

Clinical studies are still debating on the long-term benefits and consequences of endoscopic procedures that expand a stent through a tumor, leading to its compression. Herein, due to the development of magnetic tumor spheroids as magnetically deformable models of a mature tumor, we evidence that anisotropic tumor compression can trigger an increase in vitro of cancer cell proliferation and can induce in vivo amplified malignancy. It confirms other evidence of the impact of a mechanical compression on the metastasis ability and invasion potential of cancer cells.

**Abstract:**

A growing tumor is submitted to ever-evolving mechanical stress. Endoscopic procedures add additional constraints. However, the impact of mechanical forces on cancer progression is still debated. Herein, a set of magnetic methods is proposed to form tumor spheroids and to subject them to remote deformation, mimicking stent-imposed compression. Upon application of a permanent magnet, the magnetic tumor spheroids (formed from colon cancer cells or from glioblastoma cells) are compressed by 50% of their initial diameters. Such significant deformation triggers an increase in the spheroid proliferation for both cell lines, correlated with an increase in the number of proliferating cells toward its center and associated with an overexpression of the matrix metalloproteinase−9 (MMP−9). In vivo peritoneal injection of the spheroids made from colon cancer cells confirmed the increased aggressiveness of the compressed spheroids, with almost a doubling of the peritoneal cancer index (PCI), as compared with non-stimulated spheroids. Moreover, liver metastasis of labeled cells was observed only in animals grafted with stimulated spheroids. Altogether, these results demonstrate that a large compression of tumor spheroids enhances cancer proliferation and metastatic process and could have implications in clinical procedures where tumor compression plays a role.

## 1. Introduction

Cancer comes from continuously dividing cells. In order to grow, the tumor mass has to deform and disrupt the organization of the surrounding healthy tissue, which in return, resists its expansion. This growth gives rise to a constantly evolving mechanical map. Upon growth, the tumor is subjected to mechanical stress generated both by an increase in cell density and extracellular matrix (ECM) stiffening. In parallel, the surrounding tissue applies a compressive mechanical resistance toward the center of the tumor, whereas stress is tensile on the periphery. The growth-induced stress is stored within the tumor as strain energy, and tumors display an increased stiffness compared to normal tissue. For instance, in breast cancers, stiffness increases from ~3 kPa in normal tissue to ~40 kPa in high-grade carcinoma [1,2]. Additionally, cancer progression is associated with angiogenesis, and tumor expansion can trigger the collapse of surrounding blood and lymphatic vessels, can lead to an increase in blood flow resistance and an accumulation of interstitial fluid, and can ultimately cause additional mechanical stresses [3]. Solid stresses were thus quantified on breast, brain or colorectal tumors in the 0.01–0.2 kPa range [4]. Tumors may also experience mechanical constraints through surgical and/or instrumental procedures. For instance, the introduction of a colon self-expanding metal stent (SEMS) is considered an emergency alternative to avoid surgery in obstructive colon cancer and to improve the patient’s recovery [5]. The endoscopic procedure consists of expanding the stent through the tumor to re-open the colon lumen, effectively leading to compression of the tumor. While SEMS insertion leads to immediate therapeutic improvement [6], the long-term benefits are still questioned [7]. In particular, SEMS implantation in mice developing obstructive colon cancer resulted in a decrease in the survival time for treated animals [8], thus hinting at a possible mechanical SEMS-dependent stimulation of cancer progression.

The impact of mechanical stress on cancer progression has been recently *explored* in vitro for cancer cell suspensions [9] and tridimensional tumor models [10,11,12] subjected to mechanical compressions, with stresses ranging from 50 Pa to a few kPa, corresponding to the ones experienced in vivo during tumor growth. Isotropic or anisotropic confinements of small growing spheroids (less than 100 µm in diameter) led to a limitation of the final spheroid volume and were associated with an increase in cell apoptosis and a decrease in cell proliferation toward the center of the confined spheroids [13,14], the latter of which can be related to an inner pressure rise [15,16]. Controversially, anisotropic confinement performed on larger spheroids (300 µm in diameter) highlighted an increase in proliferating cells toward the spheroid center and correlated with a change in the hypoxia map and a blockage of the mitotic cycle [17]. In addition to changes in proliferative behavior, cancer cells exhibit mechanical and cell–cell adhesion properties that differ from their healthy counterparts [18].

Tridimensional spheroid models remain the simplest in vitro configuration to mimic in vivo solid tumors [19,20,21,22,23]. Compared to typical 2D culture models, tridimensional models such as spheroids recapitulate more closely the intrinsic tissue properties such as cell-cell interactions, ECM synthesis, and tumor growth kinetics [24]. Furthermore, their drug response resembles that of in vivo tumors [25,26]. Lastly, spheroids show the typical hypoxia gradient seen in tumors and allow co-culture of cancer cell lines that mimic tumor heterogeneity [27]. Taken together, these qualities make spheroid models powerful tools in the studying of cancer progression. Methods for spheroid formation include the use of micro-wells and hanging drop or centrifugation, and they are all based on locally increasing the cell density of an initially small number of cells (1000−10,000 cells) [28]. Upon cell proliferation, multicellular spheroids are formed within a few days and present diameters in the range of 250–500 µm. Increasing the spheroid size translates to a limited diffusion of oxygen and nutrients [29], and thus typical spheroid sizes remain far from those of mature tumors. Therefore, assessing the effect of mechanical compression on larger spheroids remains a challenge, which requires the development of alternative tools, first to create mature-sized spheroids without necrosis and then to subject them to controlled forces.

Current methods to mechanically stimulate tumor spheroids rely on confinement and include hydrogel embedment [30], microfluidic encapsulation [13], confinement between rigid-walls [17] or the use of osmotic pressures [14]. To the best of our knowledge, experimental tools to apply high deformations on a spheroid without direct contact and without surface forces application have not been explored.

Herein, we propose a magnetic approach to form and to compress large spheroids of mouse colon cancer cells (CT26 cell line). Iron oxide nanoparticles are internalized by CT26 cells via the endocytosis pathway. The magnetism granted to each cell then allows one to magnetically mold tumor spheroids and to compress them through the remote use of a permanent magnet. Here, we show that such a magnetic compressive stimulation triggers spheroid proliferation in vitro. Using this pre-compressed magnetic spheroid model, we then set out to explore whether this effect on proliferation was maintained in vivo after spheroid injection, where an increase in metastatic potential was observed.

## 2. Materials and Methods

### 2.1. Cell Culture and Magnetic Labeling

The mouse colon carcinoma cell line (CT26 purchased from ATCC Ref CRL-2638) was used as a model of colon cancer cells. The cells were cultured in Dulbecco’s modified Eagle’s medium (DMEM, Gibco, Thermo Fisher Scientific, Illkirch-Graffenstaden, France), supplemented with 1% penicillin–streptomycin (P/S, Thermo Fisher Scientific, Illkirch-Graffenstaden, France) and 10% fetal bovine serum (FBS, Gibco, Illkirch-Graffenstaden, France). Magnetic nanoparticles (PHENIX, UMR 8234, Sorbonne University, Paris, France) consisted of a maghemite core with a diameter of 8 nm (polydispersity index of 35%) stabilized by citrate surface coating [31,32]. In brief, they were synthesized by iron salts co-precipitation and then oxidized in a boiling solution of ferric nitrate (0.8 M) to ensure that the core composition was maghemite (γ-Fe_2_O_3_). They were then redispersed in water and functionalized with citrate anions. As a consequence, their zeta potential was found at (35 ± 5) mV. Their saturation magnetization is in the range of 55 ± 5 emu/g. They were incubated with the cells overnight in supplemented DMEM at an iron concentration of Fe = 0.5 mM. Cells then incorporated an iron mass of about 11 pg per cell, with a standard deviation among the cell population of about 35%. Other incubation concentrations are shown in Figure S1, and Figure S2 demonstrates that the magnetic labeling had no impact on cell viability and proliferation.

### 2.2. Magnetic Molding of CT26 Spheroids

**Figure 1:**
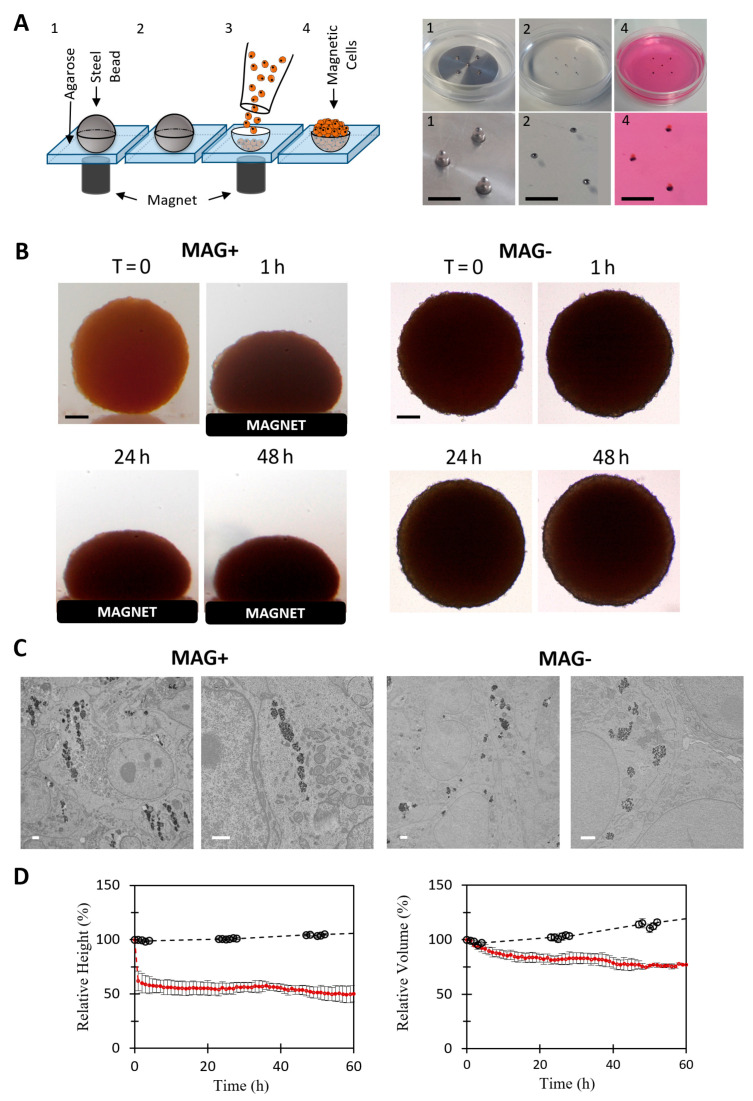
Magnetic formation (molding) and compression of CT26 spheroids. (**A**) Principle of spheroid magnetic molding, depicted with a scheme on the left and photographs on the right. Agarose molds (1 mm) were made using 1 mm magnetic beads (pictures 1 and 2). About 200,000 magnetically labeled CT26 cells were deposited on top of the molds and were attracted and aggregated within them by the cylindrical magnets placed below each mold. Aggregates were then matured overnight (picture 4). Scale bar = 5 mm. (**B**) Representative pictures of two molded aggregates. On the left, the spheroid was placed on a top of a permanent magnet (MAG+ condition). On the right, the spheroid grew without external stimulation (MAG− condition). For both, time-lapse images are shown. Scale bar = 200 µm. (**C**) Transmission electron microscopy of MAG+ and MAG− spheroids. The magnetic endosomes of the MAG+ spheroids are aligned along the field gradient, whereas in the MAG− condition, the endosomes containing nanoparticles are homogeneously distributed within the cell cytoplasm. Scale bar = 1 µm. (**D**) Temporal evolution of the relative height (left panel) and relative volume (right panel) of MAG+ (red curve, *n* = 9) and MAG− spheroids (black curve, *n* = 2). The magnetic compression results in a quick decrease in MAG+ relative height (50% after 2 h). The relative volume was stable for the first hour, followed by a slow decrease in the relative volume (25% after a few hours). Both the relative height and volume of the MAG− spheroids increased slowly.

A 2% agarose (Type I-B, A0576, Sigma-Aldrich, St. Quentin Fallavier, France) solution was prepared and heated until boiling and homogenization. A volume of 2.8 mL was deposited in a petri dish (TPP), and five 1.0 mm stainless steel beads (CIMAP) were attracted within the liquid agarose by a network of five 6 × 2 mm cylindrical magnets (Supermagnet) placed beneath the dish (Figure 1A). Agarose gelation then occurred in a few minutes and semi-spheroidal molds were ultimately obtained by removing the beads. Labeled CT26 was detached from a T75 flask, centrifuged and suspended in supplemented DMEM. About 200,000 magnetic cells were seeded inside each well, using exactly the same magnetic configuration as during the formation of the molds. Here, the magnet is only used to attract the cells within the well and is left for 5 min only. The dishes further filled with supplemented DMEM were then incubated overnight in an incubator to allow spheroid formation. The resulting spheroids were removed from the wells the day after (further denoted day 1, corresponding to the end of the formation process). Medium was then changed on a daily basis.

### 2.3. Magnetic Compression

For spheroid magnetic compressions, 6 × 6 mm cylindrical permanent magnets (Supermagnet, B = 520 mT, gradB = 180 T·m^−1^) were glued (Silicon glue, LOCTITE SI 5398, Radiospares, Boulogne-Billancourt, France) at the bottom of sterile non-adhesive Petri dishes (Grenier bio-one, 627102). Spheroids were seeded on top of the magnet (one spheroid per dish) directly after overnight formation (day 1). They were then left for 2 to 3 days (respectively day 3 or day 4) and later called MAG+ spheroids. The same number of spheroids was seeded in the non-adhesive Petri dishes without magnets and left free within (one spheroid per dish as well), further named MAG− spheroids. To quantify the deformation of the MAG+ spheroids, they were placed inside a thermally controlled homemade non-adhesive rectangular dish, allowing to camera-capture both the side and the top views of the spheroid. The permanent magnet was positioned right below the dish in the same configuration as for the Petri dishes. The compression of the MAG+ spheroids was video-monitored with a Canon camera, at 1 frame every 10 s for the first minutes, then 1 frame every 5 min the next 10 min, and 10 frames every 10 min until 1 h of deformation. Next, the deformation was video-monitored at 1 frame per hour for 60 h. The deformation was symmetrical along the axis of the magnetic gradient. The volume of the MAG+ spheroids was thus retrieved using the Guldin theorem: V = 2πS_1/2_d, with S_1/2_ the area of the semi-surface and d the distance between the center of mass of the semi-surface and the rotation axis of the spheroid. The sizes of the MAG− spheroids were measured every hour under microscope (LEICA, Wetzlar, Germany) using a 4 × objective. The MAG− spheroids are spherical in shape, with a volume V = 4πR^3^/3, where R is the spheroid radius. The global magnetization of the spheroids changed over the experimental timeframes, for both MAG− and MAG+ spheroids.

### 2.4. Alamar Blue Metabolic Assay

The metabolic activity of the MAG+ and MAG− spheroids was quantified using the Alamar Blue assay [33]. For both conditions, the assay was performed 2 and 3 days after spheroid formation (days 3 and 4, respectively) on single spheroids. The Alamar Blue reagent was incubated (10% in DMEM) with each spheroid for 3 h (100 μL per well) and was read with a fluorescence plate reader (Enspire, Perkin Elmer, Villebon-sur-Yvette, France) at 570 nm excitation wavelength and 585 nm detection wavelength following the vendor’s standard procedure in 96-well plates. A single spheroid was measured for all instances, with an incubation time with the reagent set at 2 h, and the fluorescence was recorded with an EnSpire^®^ Multimode Plate Reader (PerkinElmer, Villebon-sur-Yvette, France) using a fluorescence excitation and emission wavelength of 570 and 585 nm, respectively.

### 2.5. Cryosectioning and Immunofluorescence

Spheroids were fixed with paraformaldehyde (PFA, 4% in PBS, J61899, Alfa Aesar, Tewksbury, MA, USA) for 1h at room temperature (RT) and then conserved in PBS at 4 °C. MAG+ spheroids were fixed in the presence of the magnet. For cryo-sectioning, they were embedded in Optimal Cutting Temperature compound (O.C.T. compound, 361603E, VWR Chemicals, Rosny-sous-Bois, France) for 1h at RT, then frozen in iso-Pentane (GPR Rectapur, 24872.260, VWR, Rosny-sous-Bois, France), cooled down in liquid nitrogen, and stored overnight at −20 °C. The day after, 20 µm cryosections were cut at the center of the spheroids, parallel to the direction of the magnetic compression for the MAG+ spheroids. For immunofluorescence processing, cryosections were permeabilized with Triton (Triton X−100, dilution 1:1000 in PBS, Sigma-Aldrich, St. Quentin Fallavier, France) for 15 min at RT. Non-specific interactions were blocked using a solution of 5% bovine serum albumin (BSA, Sigma Life Science, 05479, St. Quentin Fallavier, France) in PBS for 1h at RT. MMP9 protein was labeled using primary rabbit polyclonal anti-MMP9 (ab38898, Waltham, MA, USA, dilution 1:200 with 1% of BSA, overnight at 4 °C,), and cleaved caspase−3 was labeled using monoclonal rabbit anti-Cleaved-Caspase−3 antibody (Cell Signaling, Saint-Cyr-L’École, France, 5A1E, 1:200 with 1% BSA in PBS, overnight at 4 °C). Both were coupled with anti-rabbit secondary antibody (Molecular Probes, Eugene, Oregon, USA, Alexa Fluor 488 goat anti-rabbit IgH (H + L), A11008, dilution 1:200 with 1% BSA in PBS, 2 h at RT). Ki67 protein was labeled using rabbit polyclonal anti-Ki67 antibody (Abcam, Cambridge, UK, Ab15580), coupled with an anti-rabbit Polymer Detection System (Leyca, Novolink™ DAB (Polymer) kit, Nanterre, France). Nuclei were labeled using 4′,6-diamidino−2-phenylindole (DAPI, Sigma-Aldrich, St. Quentin Fallavier, France, D9564, 1 µg/mL in PBS, 30 min at RT). All samples were mounted (Fluoromount Aqueous Mounting Medium, F4680, Sigma-Aldrich, St. Quentin Fallavier, France) and stored at 4°C after gelation of the mounting medium.

### 2.6. Microscopy and Image Analysis

Microscopy was achieved using spinning-disk confocal microscopy (Olympus, France, JX81/BX61 device, Yokogawa CSU device spinning-disk microscope (Andor Technology, Belfast, UK), 60×/1.42 oil objective). Images were processed using ImageJ open source software (Version 2.0.0-rc−68/1.52i). The stitching of the images was performed using the “Grid/Collection Stitching” plugin.

**Figure 2:**
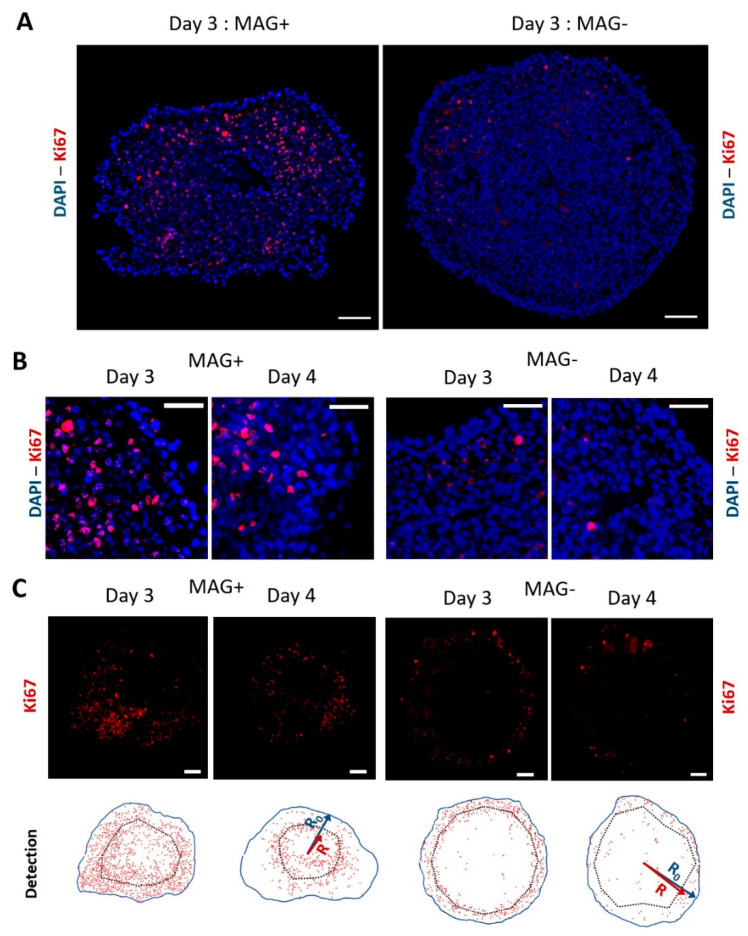
Ki67 labeling and detection at day 3 and day 4 for MAG+ and MAG− spheroids. (**A**) Typical fluorescence staining of compressed (MAG+) or left free (MAG−) CT26 spheroids for two days. The nuclei are labeled in blue (DAPI), and the proliferation marker Ki67 is shown in red. The proliferative nuclei of the MAG− spheroids are located on the edges, whereas the images evidence proliferation toward the center of the MAG+ spheroid. Scale bar = 100 µm. (**B**) Close up images at the edges of MAG+ and MAG− spheroids cultured for two and three days after formation (day 3 and day 4, respectively). Nuclei are labeled in blue and Ki67 in red. Scale bar = 50 µm. (**C**) Other Ki67 fluorescence images (in red) at day 3 and day 4 for MAG+ and MAG− spheroids. The bottom analysis shows typical detection of the Ki67-positive nuclei for the different conditions. The red dots represent the Ki67-positive nuclei, the blue line the edges of the spheroid (distance from center R0), and the dashed line represents the average localization of the Ki67-positive nuclei (distance from center R) for each sample. Scale bar = 100 µm.

The detection and the localization of the Ki67-positive nuclei were performed using the “TrackMate v.3.8.0” plugin (Gaussian Log detection). The quantification of the normalized distance <R/R0> was achieved using Matlab (MATLAB_R2016a (9.0.0.341360), License Number: 830200). Briefly, for each cryosection, the coordinates (X,Y) of the boundaries and of the center of the samples were measured using the Image J selection tool. Using the coordinates detected for each Ki67-positive nucleus, a vector corresponding to the distance between the Ki67-positive nucleus and the sample center was created (R in Figure 2). The R0 distance corresponds to the local radius of the spheroids. <R/R0> corresponds to the ratio of the two norms. The corresponding distributions then represent the percentage of Ki67-positive nuclei in the defined ring centered around the announced value of <R/R0 >, and integration over all values provides 100%. To quantify the ratio of proliferating cells, the total number of nuclei was assessed by measuring the density of nuclei. The nucleus density was multiplied by the sample surface, leading to the total number of nuclei. The nucleus density was quantified on cryosections. For each cryosection, the number of nuclei inside 5 to 10 chosen area was quantified. For each sample, the resulting nucleus density is the average density of the chosen areas. Two regions of the cryosections were addressed separately: the center (spheroid core) and the periphery (40 µm, first fourth cell layers).

To quantify the radial expression of MMP−9 (DMMP−9, example shown in Figure S5), each cryosection image was virtually divided in 10 areas, separated by a π/5 angle. For each angle, DMMP−9 was defined as the maximal radial localization from the edge of the sample to its center.

The number of cells positive to cleaved caspase−3 was quantified manually. Again, the ratio of positive cells was assessed by measuring the average nucleus density of each sample, allowing us to measure the total number of nuclei on the cryosection.

### 2.7. Transmission Electron Microscopy

MAG+ and MAG− spheroids were fixed 2 days after formation upon magnet application for the MAG+ spheroids and were processed for TEM analysis. Fixation was performed in 5% glutaraldehyde diluted in 0.1 M cacodylate buffer for 1 h and washed with cacodylate buffer. Samples were next contrasted with Oolong Tea Extract (OTE) 0.5% in 0.1 M Na cacodylate buffer, post-fixed with 1% osmium tetroxide containing 1.5% potassium cyanoferrate, dehydrated in ethanol (30% to 100%) and embedded in epoxy resins. Ultrathin sections (70 nm) were observed with a Hitachi HT 7700 TEM operating at 80 kV.

### 2.8. In Vivo Experiments

Five-week-old murine, hepatitis virus-free, and immunocompetent BALB/c females weighing 20 ± 0.5 g (Charles River, Arbresle, France) were housed in a specific pathogen-free compliant animal facility and were first acclimated for two weeks.

MAG− and MAG+ spheroids were cultured for two days after formation respectively without or with magnet application. To ensure the integrity of the spheroids as well as the absence of additional mechanical stress, the injection was performed using an enlarged tip instead of a syringe. The spheroids were easily manipulated by gently aspiring them inside the pipette. For both conditions, 10 spheroids were suspended in 200 µL of supplemented DMEM. Under anesthesia, a small incision (close to 1 cm) was performed in the middle of the abdomen of each mouse, and 10 spheroids per mouse were deposited inside the peritoneal cavity. After the injection, the peritoneum was stitched and the wound was closed with surgical staples. Mice were sacrificed 13 days after the injection, and the Peritoneal Carcinomatosis Index (PCI) was quantified using a scoring system as proposed by Klaver et al. [34]. In brief, the abdominal cavity was opened and virtually divided into 9 distinct regions (from 0 to 8). The guts represent 4 additional regions. A score from 0 to 3 was allocated for every region, and the sum of the 13 scores gave the total PCI for each mouse. A score of “0” corresponds to a complete absence of cancer nodules; “1” corresponds to 1 to 2 nodules in the region, with cumulative sizes from 1 to 2 mm; “2” corresponds to 1 to 2 nodules, with cumulative sizes from 2 to 4 mm. Finally, a score of “3” corresponds to nodules over 4 mm or more than 10 nodules. A PCI below 10 represents a limited carcinomatosis, a PCI between 11 and 20 represents an intermediate carcinomatosis and an advanced carcinomatosis corresponds to a PCI above 20.

### 2.9. Statistical Analysis

All quantitative results are represented as mean value ± standard deviation (STD). The Wilcoxon rank sum test was used for small sample numbers (*n* < 5). For a larger number of samples, the homogeneity of the variance was tested and the corresponding two-tailed Student’s *t* tests were performed. In particular, for quantification of the MMP−9 depth penetration, a Wilcoxon rank sum test was performed by pooling all the angular radius measured for a same condition. *p* value was used to indicate the statistical significance of the results (* *p* < 0.05, ** *p* < 0.01, *** *p* < 0.005).

## 3. Results

### 3.1. Cancer Spheroid Magnetic Formation and Compression

Magnetic labeling of mouse colon cancer cells (CT26) with iron oxide nanoparticles upon overnight incubation at Fe = 0.5 mM provided each cell with a magnetic moment at saturation of about Mcell = 7 × 10^−13^ A·m^2^, which corresponds to 11 pg of iron internalized per cell. This incubation condition was optimized to reach the highest nanoparticle load for the lowest dose. Figure S1 shows the nanoparticles cell uptake curve as a function of nanoparticles concentration, reaching saturation at Fe = 0.5 mM. The magnetic labeling had no effect on cell viability and proliferation (Figure S2). Upon exposure to the permanent magnets used in the study (B = 520 mT; gradB = 180 T·m^−1^), each magnetic cell experienced a magnetic force in the order of 120 pN.

To form the magnetic tumor spheroids, a magnetic molding procedure was implemented, as presented in Figure 1A. As described in detail in the Methods section, it consists of magnetically attracting labeled cells inside 1 mm diameter agarose molded spherical wells. This molding-assisted magnetic aggregation method delivers overnight tumor spheroids with a well-defined diameter of 878 ± 54 µm (Figure 1B). No necrosis was observed at the spheroid center after 48 h of culture (Figure S3).

The spheroid magnetic moment was measured by magnetometry, with an average value of Mspheroid = (1.3 ± 0.3) 10^−7^ A·m^2^ at magnetic field saturation. The application of the permanent magnet then resulted in a magnetic force (M.gradB) on each spheroid in the range of 25 µN. This force is a volume force, such as gravity, and was close to 7 × 10^4^ N·m^−3^, which is equivalent to about 90 g. This magnetic gravity is important enough to deform the spheroids but not to damage the cells and is below routine cell culture centrifugation, at 100–300 g.

Figure 1B shows typical spheroids up to two days after their formation, either subjected constantly to a permanent magnet (MAG+) or left free without a magnet (MAG−). It evidences the large anisotropic magnetic deformation of the MAG+ spheroids. At the cell level, the intracellular localization of the magnetic nanoparticles was observed by transmission electron microscopy (TEM, Figure 1C). As expected, for both MAG− and MAG+ conditions, the nanoparticles are stored within endosomes, which are spread homogeneously inside the cells for the MAG− condition. For the MAG+ condition, the magnetic force created by the magnetic field gradient and acting on a single magnetic endosome, in the 0.1 pN range, was not sufficient to aggregate the endosomes on the cell membrane toward the magnet side. However, the magnetic dipolar force created by the high-intensity magnetic field and attracting endosomes one to another, in the 100 pN range when in contact, produced a well-defined alignment of the magnetic endosomes alongside the direction of the magnetic field.

Finally, the relative dimensions of the spheroids (height or volume relative to initial values) were quantified over time, and the average values are shown in Figure 1D. It demonstrates that the MAG+ spheroids experienced a two-step deformation: first an important and rapid deformation (40% decrease in height within the first 20 min), and then a second almost linear decrease to reach 50% of the initial height. The kinetics of the rapid compression are shown more precisely in Figure S4. The MAG+ spheroid volume did not vary during the initial deformation, but it then slightly decreased afterward down to 75% of its initial value. Comparatively, both the diameter and volume of the MAG− spheroids slightly increased over time, with a 3% increase in diameter and 10% increase in volume.

### 3.2. Magnetic Spheroid Compression Increases Cell Proliferation and Metalloproteinase MMP−9 Expression In Vitro

MAG− and MAG+ spheroids were stained for the proliferation marker Ki67 48 h and 72 h after their formation (corresponding to three days and four days of spheroid maturation). Representative images are shown in Figure 2, with DAPI-labeled nuclei in blue and the Ki67 antibody in red. Figure 2A shows the whole spheroids at day 3, and Figure 2B closes up on the periphery region at day 3 and day 4. Qualitatively, the cells with a nucleus positive to Ki67 appear more present at the center for the MAG+ spheroids, while they are mainly located at the periphery for the MAG− spheroids. To quantify this, the positions of both Ki67 positive nuclei and negative nuclei were systematically tagged, as illustrated in Figure 2C at days 3 and 4.

**Figure 3:**
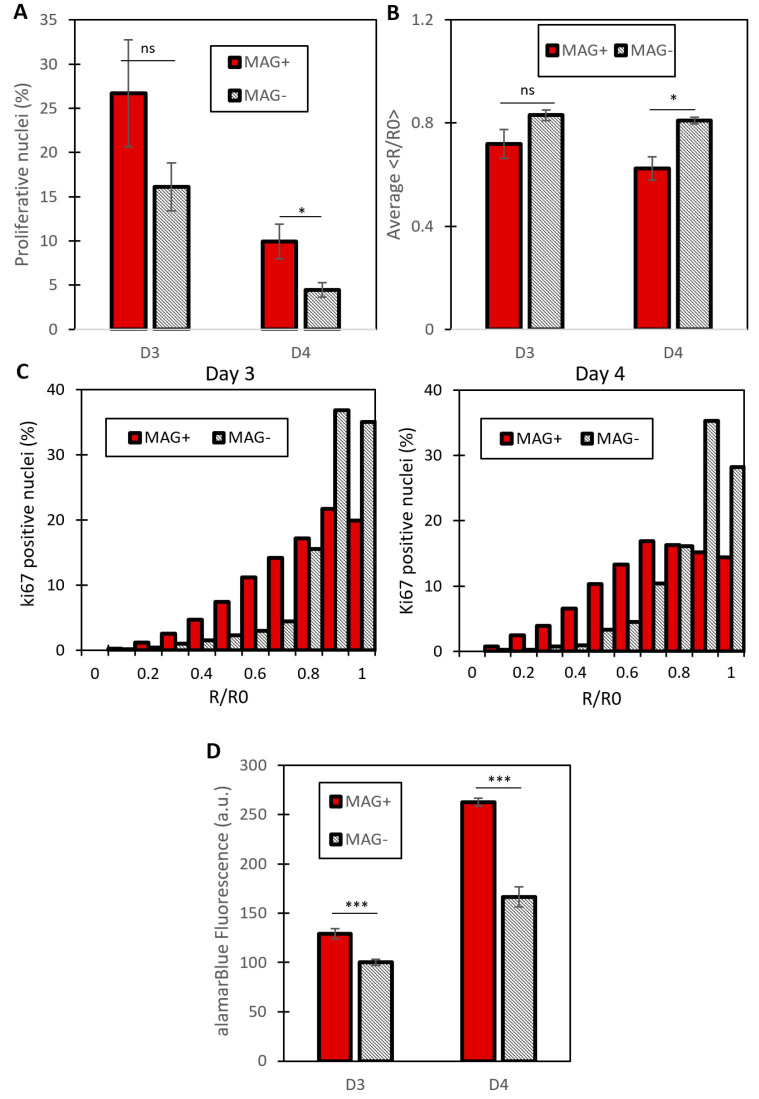
Quantification of cell proliferation of compressed (MAG+) or free (MAG−) spheroids. (**A**) Percentage of proliferating cells for MAG+ and MAG− spheroids (respectively red and grey bars) at day 3 and day 4 of spheroid maturation. MAG+ spheroids experience an increase in the number of proliferating cells for the two days. Proliferation is evaluated as the ratio between the number of Ki67-positive nuclei and the total number of nuclei (at day 3, *n* = 3 for both conditions, at day 4, *n* = 3 for MAG−, *n* = 5 for MAG +, * *p* < 0.05). (**B**) Average of the normalized distance <R/R0> for the two conditions at the two maturation times (days 3 and 4). This distance represents the localization from the spheroid center of a Ki67 positive nucleus. This decreases for the MAG+ spheroids, demonstrating an increase in the proliferative nuclei toward the MAG+ spheroid center (*n* = 3 spheroids for both conditions at day 3; *n* = 3 and *n* = 5 spheroids at day 4 for MAG+ and MAG− conditions, respectively; *p* < 0.05). (**C**) Cumulative distributions of the normalized distance <R/R0>. To obtain these distributions, the Ki67 localizations were pooled for every image of a single condition. It demonstrates a shift of the proliferation toward the center of the MAG+ spheroids, whereas the MAG− distribution is grouped around 1, which represents nuclei localized at the edge of the spheroid (at day 3, *n* = 1915 Ki67-positive nuclei for the MAG+ spheroids, *n* = 2653 Ki67-positive nuclei for the MAG− spheroids, *p* < 0.005; at day 4, *n* = 937 Ki67-positive nuclei for the MAG+ spheroids, *n* = 2359 Ki67-positive nuclei for the MAG− spheroids, *p* < 0.005). (**D**) Metabolic Alamar Blue assay. The relative fluorescence measured is shown for MAG+ and MAG− spheroids (red and striped bars, respectively) for day 3 and day 4. For both times, the proliferation of MAG+ spheroids is increased compared to MAG− spheroids (at day 3, *n* = 15 for the MAG− spheroids and *n* = 26 for the MAG+ spheroids, *** *p* < 0.005, at day 4, *n* = 15 for MAG+ and *n* = 14 for MAG−, *** *p* < 0.005).

The percentage of proliferating nuclei, as well as their localization, could then be quantified (Figure 3). The proportion of proliferating nuclei decreased between 48 and 72 h for both MAG− and MAG+ conditions. As detected in Figure 3, proliferation decreased between day 3 and day 4, which is due to the fact that the spheroids are millimeter-sized, such that they stop growing at some point, as demonstrated in previous work [22].

On both day 3 and day 4, the MAG+ spheroids exhibited an increase in the proportion of proliferating nuclei as compared to the MAG− spheroids (Figure 3A). To evaluate the position of the proliferating cells, the normalized distance <R/R0> was introduced (see Figure 2C for illustration), with R being the distance from the center of the spheroid of any Ki67-positive nucleus and with R0 being the distance from the center to the edge of the spheroid at the same angle (equivalent to the local spheroid radius). Therefore <R/R0> stands for the normalized local position of any Ki67-positive nucleus. Figure 3B shows the average <R/R0> for both conditions at days 3 and 4. It is lower for the MAG+ spheroids, revealing a shift of the proliferation toward the center of the spheroids, triggered by the magnetic compression. It is even clearer on the <R/R0> cumulative distributions shown in Figure 3C. More than 70% of <R/R0> values are between 0.8 and 1 for the MAG− spheroids, which corresponds to the periphery of the spheroids. By contrast, for the MAG+ spheroids, the <R/R0> values are distributed almost equally throughout the spheroids (linear increase in the cumulative distribution, as the ring surface increases linearly as well). The magnetic compression also enhanced the invasion of the cancer cells (Figure S6), although this should be taken with care because the magnetic force itself also impacts the initial area of invasion.

**Figure 4:**
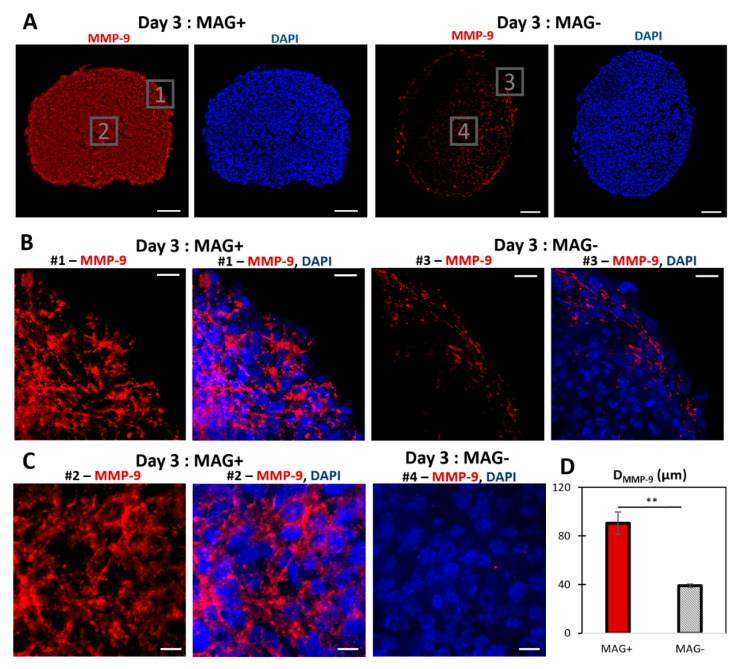
MMP−9 labeling of MAG+ and MAG− spheroids at day 3. (**A**) Typical fluorescent staining of MAG+ and MAG− spheroids cultured for two days after formation. MMP−9 is labeled in red, and the nuclei are labeled in blue (DAPI). For the MAG− spheroids, MMP−9 was mostly expressed at the edges. The MAG+ spheroids showed significant MMP−9 expression toward the center. Scale bar = 100 µm. (**B**) Close up images (# = number) at the edges of the spheroids: MMP−9 only (red) and merged images (MMP−9 in red, DAPI in blue). Scale bar = 20 µm. (**C**) Close up images for MAG+ (MMP−9 only in red and merged image with DAPI in blue) and MAG− spheroids (merged image of MMP−9 in red and DAPI in blue). Scale bar = 10 µm. (**D**) Localization of MMP−9 for the two conditions. The maximal radial MMP−9 localization, from the edges toward the spheroid center was quantified. The average MMP−9 depth was higher for the three-day MAG+ spheroids (red bar) than for the MAG− spheroids (striped bar), *n* = 2 and *n* = 3, respectively (** *p* < 0.001).

Figure 4 shows the staining of the matrix metalloproteinase MMP−9 on MAG− and MAG+ spheroids labeled at day 3. It reveals an increase in the expression of MMP−9, mostly in the center of the spheroid. The MMP−9 radial localization was quantified (Figure S5) with an average distance from the edge to the spheroids center of 90.6 ± 18.8 µm and 39.3 ± 3.9 µm for MAG+ and MAG− conditions, respectively. The upregulation of MMP−9 was confirmed by quantitative real-time PCR (Figure S7). To rule out any potential effect of nanoparticles-mediated force application on intracellular activities, control experiments were carried out on cancer cells under a magnetic field when in 2D culture (Figure S8) or organized as a loose aggregate with no 3D interaction and therefore no magnetic compaction (Figure S9). No effects of the intracellular magnetic force alone on cell metabolic activity were detected.

Finally, another series of in vitro experiments was performed with another cell line, U−87 MG (purchased from ATCC), which is derived from human glioblastoma. It was selected to verify both the cell line as well as species specificity (human versus mouse for CT26). U87 spheroids were formed by the magnetic molding process overnight using the same procedure as for CT26 spheroids. Spheroids were collected at day 1 and placed upon a magnet, resulting in a similar deformation as obtained for the CT26 spheroids (Figure S10A). The spheroids’ (*n* = 15) metabolic activities were then measured either after two days of magnet application (day 3) or one day later, with a magnet applied over the 3 consecutive days (day 4), revealing a significant increase in spheroid proliferation for the MAG+ compressed ones (Figure S10B).

### 3.3. Magnetic Compression Increases the Metastatic Potential of the Spheroids In Vivo

**Figure 5:**
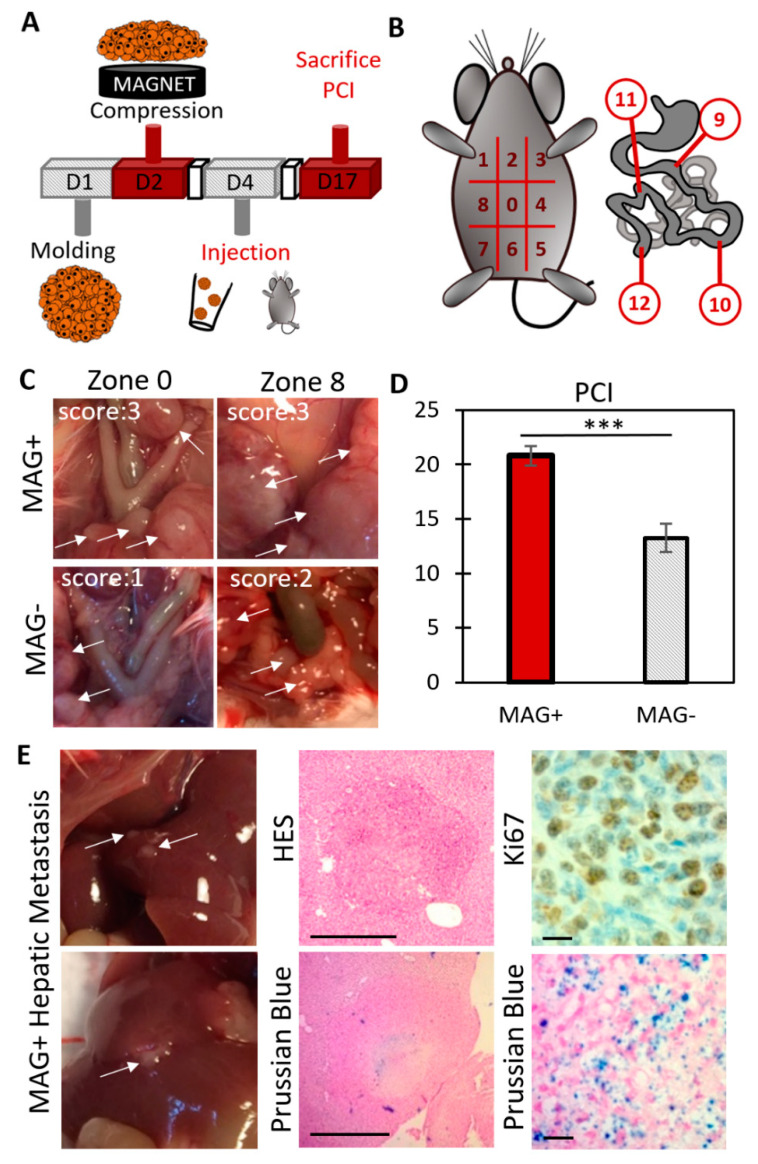
Evaluation of the carcinomatosis progression in mice injected with MAG+ or MAG− spheroids 3 days after their formation. (**A**) Overview of the experiment. Magnetically molded CT26 spheroids were cultured for two days upon magnet application (MAG+ spheroids) or without magnets (MAG− spheroids). Then, for each condition, 10 spheroids per mouse were injected into the peritoneum. Mice were sacrificed 13 days later, and the Peritoneal Carcinomatosis Index (PCI) was evaluated. (**B**) PCI evaluation: the peritoneum is divided into 13 regions. Depending on the number and size of the tumor nodules, a score between 0 to 3 is allocated for each region. The sum of the scores provides the PCI for each mouse. (**C**) Typical images during PCI analysis. The white arrows indicate tumor nodules. For the two regions of the MAG+ mouse, a score of 3 was allocated. Scores of 1 and 2 were allocated for the regions *n*°0 and *n*°8 of the MAG− mouse, respectively. (**D**) Average PCI for mice injected with MAG+ spheroids (red bar) and MAG− spheroids (striped bar). The mice injected with the compressed spheroids (MAG+) experienced an increase in cancer progression (two different experiments, in total *n* = 8 and *n* = 10 for MAG+ and MAG−, respectively, with *** signifying *p* < 0.005). (**E**) Immunohistological staining of hepatic metastasis (indicated with white arrows) in mice injected with MAG+ spheroids. The top middle picture shows hematoxylin–eosin staining (HES, cytoplasm and nucleus labeling) of a metastasis section of approximately 800 µm in diameter (scale bar = 500 µm). The bottom middle image shows a Prussian blue staining (iron labeling, scale bar = 500 µm). Iron is clearly detected at the center of the metastasis, indicating that it originates from the injected cells. The right images present a magnification of a Ki67 staining and of a Prussian blue staining. The metastases were proliferating (Ki67-positive) and originated from the injected cells (Prussian blue positive). Scale bar = 20 µm for the right images.

The in vivo experiment protocol is illustrated in Figure 5A. MAG+ and MAG− spheroids were cultured for two days after their molding, with or without the magnet application, respectively. Then, for each condition, 10 spheroids were injected into the mouse peritoneal cavity. Four to five mice were injected per condition, and the experiment was repeated twice. The mice were sacrificed 13 days after the injection, and the peritoneal cancer index (PCI) was evaluated as a read out of the cancer progression (Figure 5B). Typical images of different zones, together with associated PCI scores, are shown in Figure 5C. Average PCI values are presented in Figure 5D, corresponding to a PCI of 20.8 ± 2.7 for mice injected with MAG+ spheroids, whereas only of 13.2 ± 3.7 (*p* < 0.005) for mice injected with MAG− spheroids. Such PCI values correspond to advanced and intermediate carcinomatosis, respectively [35]. Besides, the ex vivo imaging of Ki67 on tumor nodules (Figure S11) revealed a significant increase in the percentage of proliferative nuclei for the MAG+ compressed spheroids.

Additionally, hepatic nodules (Figure S12A) were observed for 40% of mice injected with MAG+ spheroids (Figure S12B), while none could be detected within mice injected with the MAG− spheroids. As illustrated in Figure 5E (additional images are shown in Figure S12C), these hepatic tumors were Ki67-positive metastases. In addition, Prussian blue staining evidenced the presence of iron oxide nanoparticles located at the center of the hepatic metastasis, confirming that they originate from the injected MAG+ spheroids.

## 4. Discussion

Magnetic cell labeling using iron oxide nanoparticles was recently proposed as a tool to form and to mechanically stimulate cell assemblies [36,37,38,39]. The nanoparticles are internalized by the cells through the endocytosis pathway and stored inside endosomes, without interfering with cell metabolism and function, including differentiation [31,40]. Herein, the magnetism conferred to the cells due to magnetic nanoparticle incorporation was initially used to form cancer cell spheroids by magnetic molding. The magnetic molding allowed delivering 1 mm size spheroids overnight, which cannot be achieved by common spheroid formation methods. This process ends up with 1 mm diameter spheroids mimicking the carcinoma nodules obtained in vivo (clinically found between 1 mm to a few cm) [41]. No necrosis was detected at the core of the spheroids, and spheroids continued to slightly grow over the first days in the control condition (MAG−, Figure 1D).

The spheroid magnetism next served to mechanically stimulate the spheroids. The combination of a high magnetic field gradient and high spheroid magnetization resulted in spheroid compression with a 50% height decrease and an ellipsoidal shape with a 1:2 axis ratio. For such magnetic compression, the applied stress depends on the distance from the magnet, and it is driven by the upper cells pushing on the cells below them. The volume magnetic force is in the 7 × 10^4^ N·m^−3^ range, and the maximal stress is about 100 Pa.

**Figure 6:**
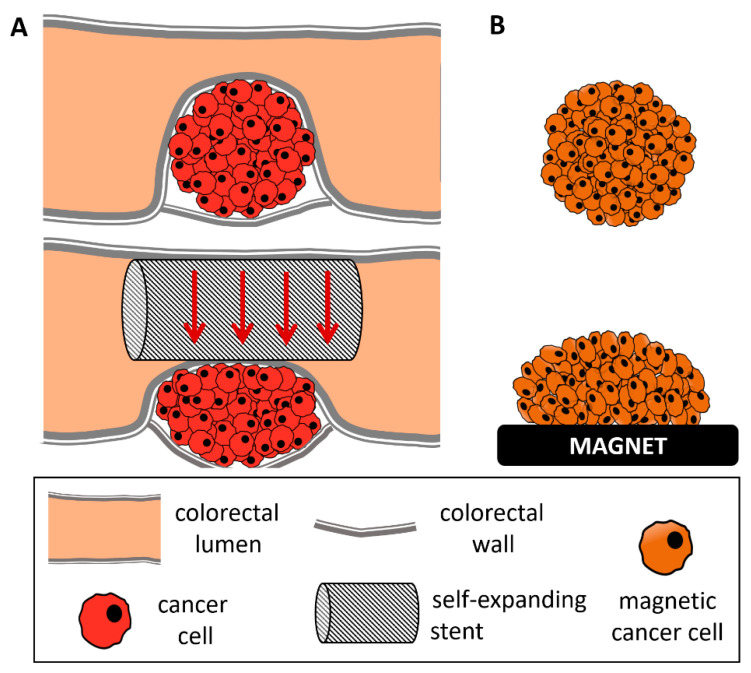
Schematic comparing an obstructive colorectal cancer treated with self-expanding stent and a magnetically compressed tumor spheroid. (**A**) Tumor growth within the colorectal wall, occluding the lumen of the colon almost entirely (**top**). To avoid this obstruction, a self-expanding stent is inserted inside the colorectal lumen and extended to obtain a re-opening of the lumen, up to 75% of its initial radius (**bottom**). This results in anisotropic compression of the tumor. (**B**) Representation of a magnetically molded tumor spheroid (**top**) and its anisotropic compression upon magnet application (**bottom**).

Other mechanical stimulation of cancer cells generally involved either smaller spheroids subjected to growth confinement [10,13,14,17,30] or direct compression. In the latter case, forces on 2D layers or scaffold embedded cells ranged from 0.05 [9] to 8 kPa [42], and up to 20 kPa when applied to spheroids [14]. Importantly, these last high pressure values corresponded to isotropic (osmotic) compression, which is different from anisotropic (uniaxial) compression. This geometry of deformation was chosen to mimic the uniaxial compression experienced with a colonic self-expending metal stent (SEMS), which is remarkably high (~50%) and corresponds to pressures of about 100 Pa. The endoscopic SEMS procedure consists of applying a mechanical constraint on one side of a colon-developed tumor in order to re-open the colon lumen. Remarkably, in this stent model in humans, the procedure is comparable with the two-step stimulation applied here, with a first strong dilatation obtained by the stent and a second less important one that takes 2 days for the stent to be fully open. As depicted in the schematics of Figure 6, the magnetic spheroid compression thus mimics the deformation experienced by the on-site tumor, where the introduction of a SEMS was evidenced to lead to a re-opening of a lumen with an obstruction of more than 75% [8].

Herein, the magnetic compression triggered an increase in the number of Ki67-positive nuclei, consistent with an increase in the cell metabolic activity. Proliferation in constrained spheroids remains a controversial issue. For spheroids compressed in all directions (isotropic deformation), Ki67 expression decreases in the core [13,14], while it can facilitate tumor invasion [43]. For a directional deformation, both a decrease [10] and an increase [17] in Ki67 have been reported. In the latter study, the final shape and size after spheroid compression (300 × 600 µm rod shape) were similar to the magnetic compression experienced here. In other studies, the decrease in proliferation could also be correlated with an important increase in cell apoptosis [8,10], with the number of caspase−3 positive cells ranging between 40% and 80% in the spheroids’ core. Herein, the caspase−3 positive cells are below 5% after 48 h of compression (Figure S3). Moreover, the delocalization of cell proliferation observed toward the spheroids’ centers appears to be associated with an increase in the expression of MMP−9. During tumor development, in association with the hypoxic environment [44] resulting from a stiffening of the stromal ECM [45,46], cancer cells experience an increased expression of MMPs, allowing them to remodel their surrounding matrix [47] and trigger a metastatic process. This correlation between MMPs and cell proliferation and metastasis has been documented in cancer spheroid models. Multicellular tumor spheroids subjected to increasing doses of MMP−1 inhibitor showed a retardation in growth [48]. A similar response was observed in cancer-associated fibroblast spheroids, where MMP−1 inhibition decreased the cancer invasion rate [49]. Furthermore, using a model of stromal cell spheroid co-culture, a decreased spheroid invasion area into collagen 1 was observed, yet cell migration was not fully eliminated [50]. In a different approach, MMP−9 secretion has been associated with increased invasion potential in a heterogeneous spheroid model of mesenchymal stem cells and head and neck squamous cell carcinoma [51]. By contrast, the role of mechanical stress on MMP expression is still open to exploration. A compression-triggered increase in MMP expression was detected in bone cancer cells [52], glioblastoma and breast cancer cells [9] yet was organized as monolayers or embedded in an ECM-like reconstituted gel. Here, the increase could be related to an enhanced metastatic potency of the cells inside the compressed spheroids, as it is clinically associated with an increase in cancer invasion and a decrease in patient prognosis [53,54,55]. While invading cells in the MAG− spheroids are limited to the first cell rows, all cells in the MAG+ spheroids could potentially invade the environment. Future experimental work could focus on elucidating the role of different key players in the metastatic process of the magnetic compression spheroid model. For instance, enabling of an invasion due to the expression of epithelial–mesenchymal transition genes [56] and how it correlates to magnetic compression could broaden the understanding of the role of how such mechanical forces play in metastasis.

Finally, the in vivo experiments led to a significant increase in the PCI index for mice injected in the peritoneal cavity with the compressed MAG+ spheroids. This peritoneal carcinomatosis index is used as a reliable read out of cancer progression [57]. Besides, several mice injected with compressed spheroids presented hepatic metastasis, with iron detected in their core, confirming that they originated from the initial spheroids. Such organ invasion also denotes an increase in metastatic potency [58], and it is probably related to the activation of mechanotransduction pathways [59,60]. For instance, in mice, the β-catenin pathway has been associated with the proliferation of cancer cells in mechanically stimulated healthy tissues [61]. Furthermore, upon uniaxial compression, such as the one experienced here, traction forces are generated in the directions orthogonal to the direction of compression, to which β-catenin should be sensitive, as demonstrated in the developmental mechanosensitive pathway [62].

## 5. Conclusions

Due to an original set of magnetic methodologies mediated by the internalization of iron oxide nanoparticles, the harmful role of physical factors in cancer progression was evidenced in vitro and in vivo. The overall results are consistent with the ones observed in mice treated with SEMSs, where a decrease in the mouse survival time was observed after tumor compression thus questioning the long-term benefits and the consequences of such a surgical act [63,64]. Herein, magnetic anisotropic tumor pre-compression triggers an increase in vitro of both cancer cell proliferation and expression of matrix metalloproteinases, and it induces in vivo amplified malignancy after injection.

## Data Availability

The data that support the findings of this study are available from the corresponding author and in the Supplementary Materials file.

## Supplementary Materials

The following are available online at https://www.mdpi.com/article/10.3390/cancers14020366/s1, Figure S1: Magnetophoresis of nanoparticles-labeled cells; Figure S2: Effect of magnetic nanoparticles internalization on cell proliferation; Figure S3: Cleaved caspase−3 labeling; Figure S4: Time evolution of compressed spheroids; Figure S5: Quantification of the MMP−9 radial localization; Figure S6: Quantitative PCR analysis of matrix metalloproteinase genes MMP9 and MMP13; Figure S7: Effect of the magnetic field gradient on cell metabolic activity; Figure S8: Impact of magnet application on loose aggregates of cancer cells; Figure S9: Formation and compression of U87 glioblastoma spheroids; Figure S10: Ex vivo tumor nodules immunohistochemical staining of Ki−67; Figure S11: Hepatic metastasis; Figure S12: A. Hematoxylin and eosin staining of two hepatic metastases at low magnification (×25) recovered from mice injected with compressed MAG+ spheroids. B. Number of hepatic metastasis detected in mice injected with control MAG− spheroids (none) and with compressed MAG+ ones, per mouse. C. Immunohistochemical staining of Ki-67 for two MAG+ spheroids derived hepatic metastases, at magnification ×400.
