# Supplementary material for: Magnetic Compression of Tumor Spheroids Increases Cell Proliferation In Vitro and Cancer Progression In Vivo
Source: Cancers (Basel). 2022 Jan 12;14(2):366. doi: 10.3390/cancers14020366 (PMC8773997; doi:10.3390/cancers14020366)
Supplement: Supplementary file 1 [file cancers-14-00366-s001.zip › cancers-1492319-supplementary.pdf]

Supplementary Materials

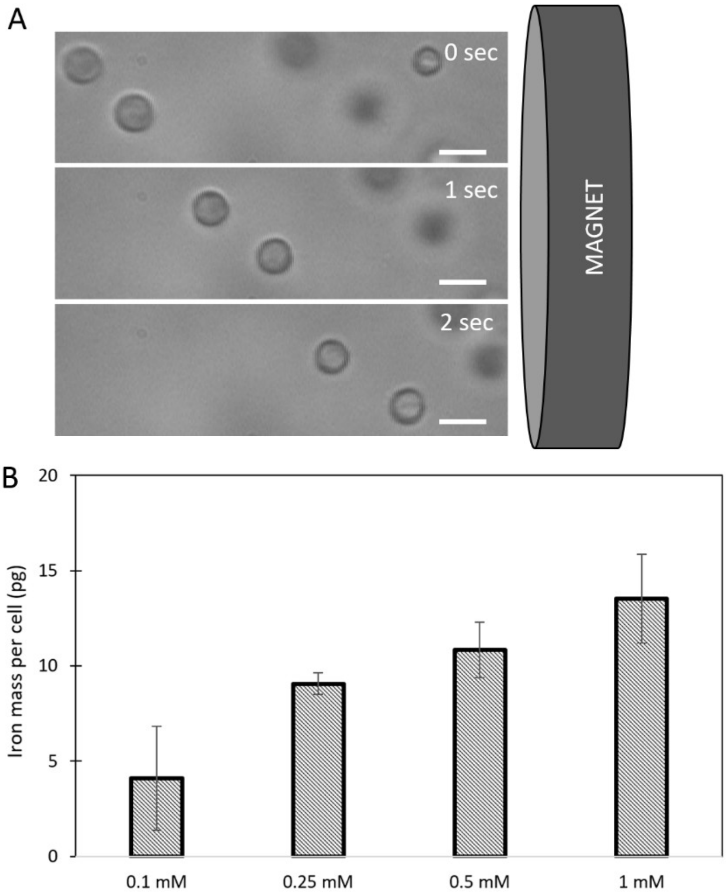

Figure S1. Magnetophoresis of nanoparticles-labeled CT26 cells. (**A**) When redispersed in culture medium after detachment, single magnetic CT26 cells are attracted toward a permanent magnet (B = 135 mT, grad(B) = 17 T.m^{-1}) by a magnetic force (F = M_{cell}.gradB), balanced by Stokes' drag. The cell velocity then directly infers the cell magnetic iron mass. Scale bar = 20 µm. (**B**) Cells were labeled with solutions presenting increasing nanoparticles concentrations during the night (18h). The cells were washed and detached to perform magnetophoresis (50 single cells analyzed per condition and per experiment). The iron load per cell is distributed among the cell population with standard deviation in the range of 35 %. The graph shows the average iron load per cell (*n* = 3).

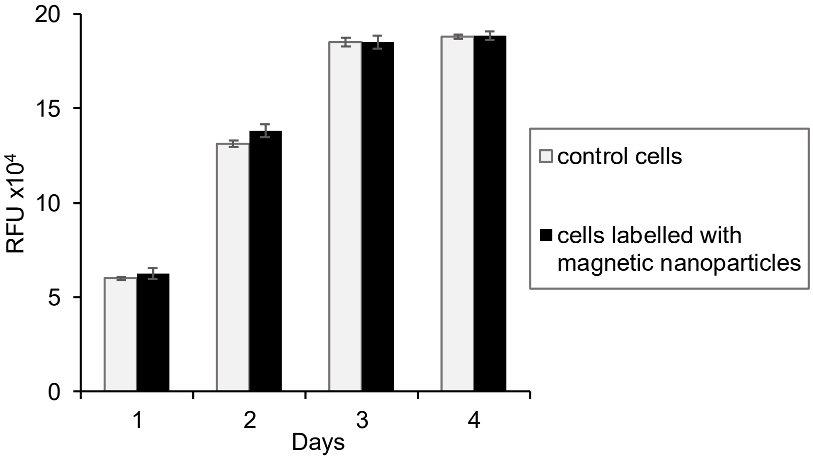

Figure S2. Monitoring of cell proliferation for control CT26 cells and CT26 cells with incorporated magnetic nanoparticles. Control cells and magnetic nanoparticles – labelled cells (typical incubation

at [Fe]=0.5 mM overnight) were detached and seeded in 96 well plate (5000 cells per well). The cells metabolic activity, directly related to the number of cells, was measured every next day, from day 1 to day 4 (n = 6 per condition). The Alamar Blue reagent was incubated with the cells for 2 hours (10% 100 μL per well), and was read with a fluorescence plate reader (Enspire Perkin Elmer) at 570 nm excitation wavelength and 585 nm detection wavelength. It evidences that the presence of nanoparticles within the cells has no impact on their proliferation behavior.

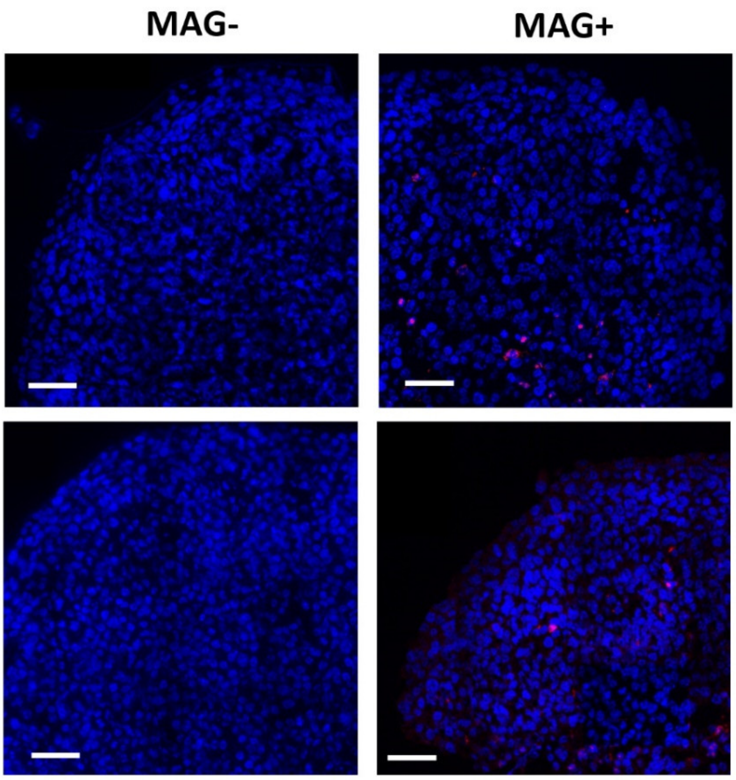

Figure S3. Cleaved caspase-3 labeling. Spheroids were fixed 48h after the magnetic molding (day 3). The figure presents CT26 spheroids at day 3 (48h after the molding), left free (MAG-, left image) or subjected to a permanent magnet (MAG+, right picture). No cleaved caspase-3 positive cells were noticed for the MAG- conditions (n = 4). For the MAG+ spheroids, less than 5% of the cells are positive to cleaved caspase-3 (4.5 ± 0.5%, n = 3). Scale bar = 50 μm.

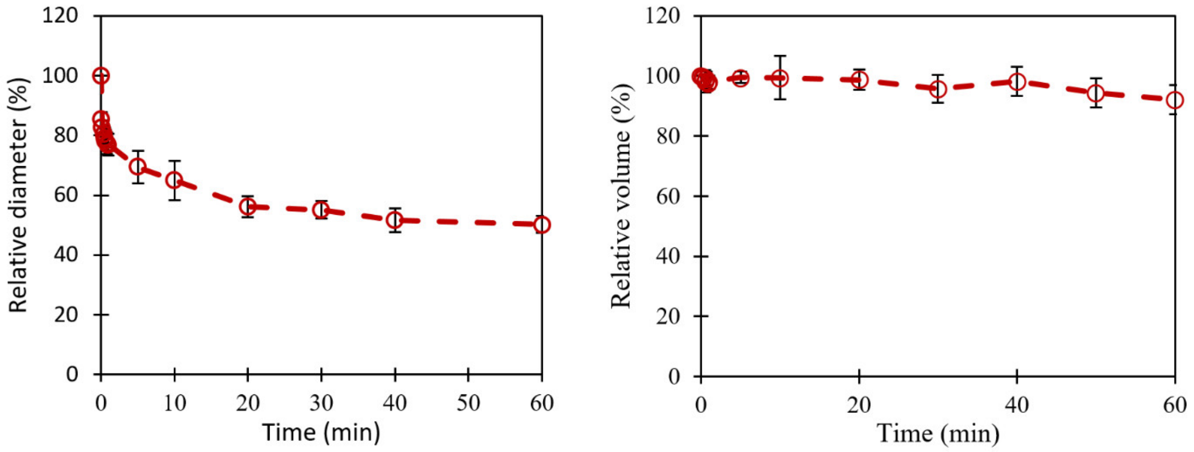

Figure S4. Time evolution of relative diameter and relative volume of compressed CT26 spheroids (MAG+) for short-time deformations. The relative diameter presented a quick decrease, whereas the volume was constant within the first hour of the deformation (n = 2).

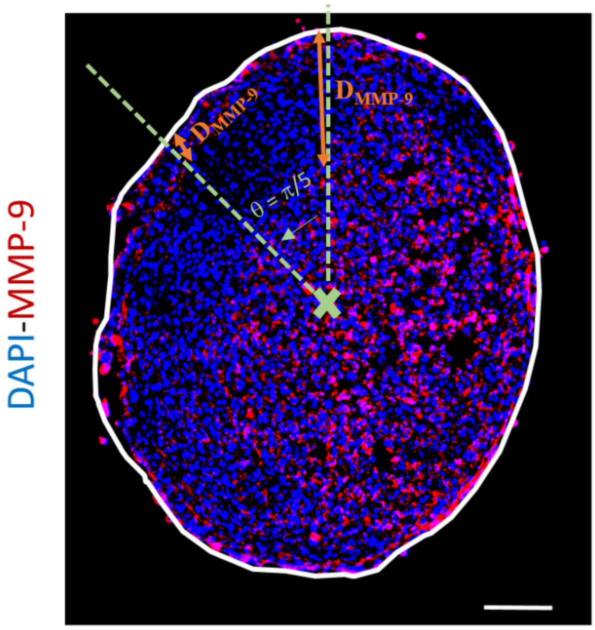

Figure S5. Quantification of the MMP-9 radial localization. A Mag- spheroid is shown and is virtually divided in 10 areas separated by a π/5 angle. For each angle, the radial distance of MMP-9 expression was measured, from the edge to the center of the sample. MMP-9 is labelled in red and the nuclei in blue (DAPI). The white line represents the sample boundary, the green lines represent the delimitation of two regions distanced of π/5. The orange arrows represent two measurements of the MMP-9 radial localization. Scale bar = 100 μm.

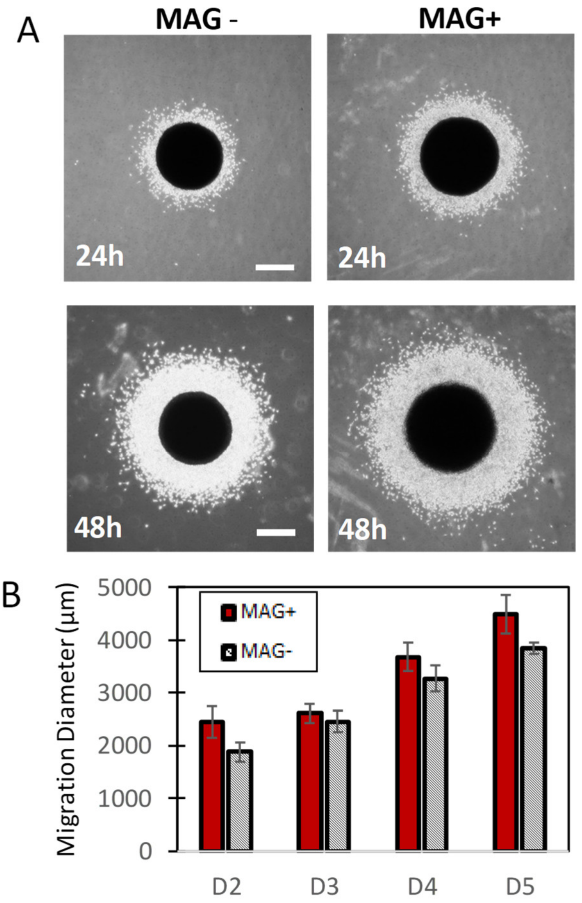

Figure S6. Migration of magnetically compressed CT26 spheroids (MAG+) and control spheroids without magnet application (MAG-). (**A**) Images obtained 24 hours and 48 hours after the seeding of the MAG- and MAG+ spheroids on adhesive surface coated with gelatin. (**B**) Average diameter of the area of cell migration at the periphery of growing spheroids over time (from day 2 to day 5).

n = 21 and n = 22 for the MAG- and MAG+ spheroids, respectively. A difference was detected, although it should be taken with care, because the magnetic force also impacts the initial area of invasion.

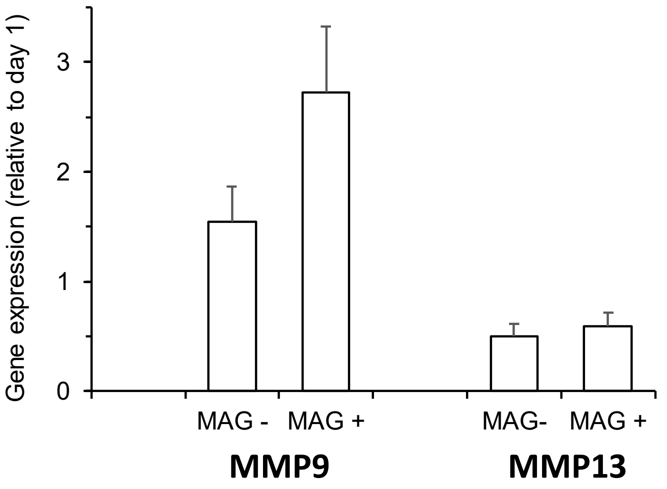

Figure S7. Quantitative PCR analysis of matrix metalloproteinase genes *MMP9* (5′- AGTGGGACCATCATAACATCACAT-3′; 3′- TCTCGCGGCAAGTCTTCAG-5′) and *MMP13* (5′- CCTGATTCTTGCGTGCTATGAA-3; 3′-CAGATGGACCCCATGTTTGC-5′) was carried out using the StepOnePlus™ System and SYBR Green as fluorophore (Life Technologies, Saint-Aubin, France) according to the manufacturer's protocol. Levels of MMPs gene mRNA were normalized to *RPLP0* gene mRNA (5′- GCCAGCTCAGAACACTGGTCTA -3′; 3′- ATGCCCAAAGCCTGGAAGA -5′), and are shown here normalized by gene expression at day 1.

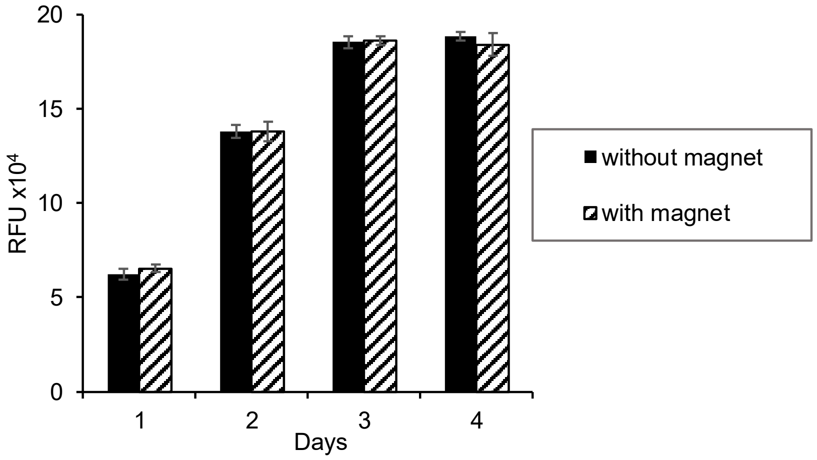

Figure S8. CT26 cells labeled with magnetic nanoparticles were seeded in 96-well plates (5000 cells per well, n = 6 per condition), positioned or not on top of the small cylindrical magnets used for flattening the spheroids. The cell proliferation was then assessed over 4 days, with the magnets left in place, by Alamar Blue measurement (10% in 100 µL of medium per well for 2 hours, followed with fluorescent reading, at 570 nm excitation and 585 nm emission). No effect of the magnetic field gradient on cell metabolic activity is detected.

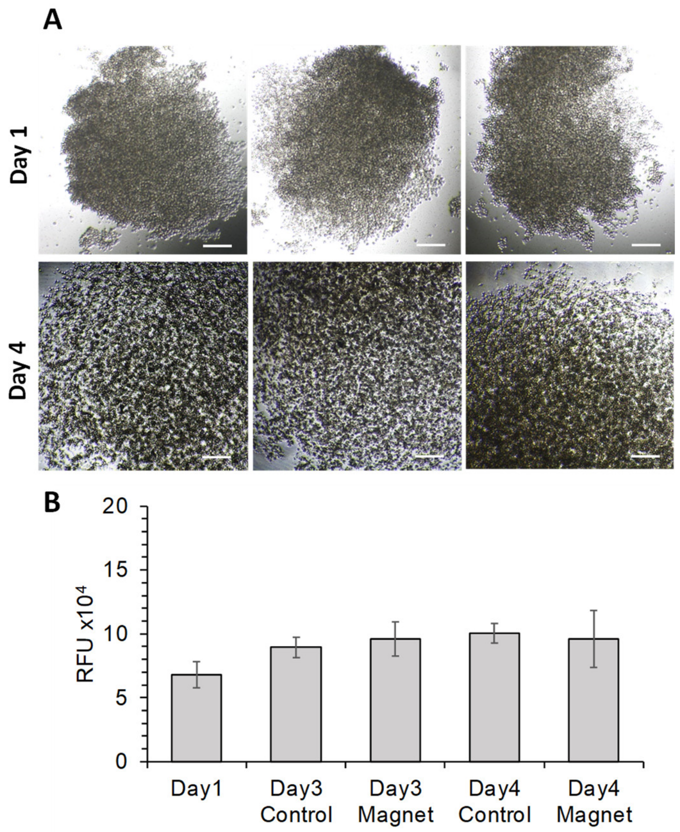

Figure S9. Impact of magnet application on loose aggregates of cancer cells. Human prostatic cancer PC-3 cells were magnetically formed in the molds with the exact same procedure as performed with CT26 cells. After overnight formation in the molds (day 1), the aggregates were placed in non-adhesive dishes, and observed with a 4× objective by optical microscopy (**A**, top), showing a loose structure rapidly dissociating, with no cell-cell cohesiveness. On day 4 (**A**, bottom), the cells have spread onto the surface, only vaguely reminiscent of the initial aggregate. The cells' metabolic activity was assessed at day 3 and day 4, without (Control) and with (Magnet) continuous application of the cylindrical magnets over the 2 or 3 days. The same protocol (Alamar Blue) that was set up for CT26 spheroid proliferation detection was used. There is no significant change in the fluorescent signal (relative fluorescent units) between the control group and the one exposed to the magnet over the measured time points (**B**).

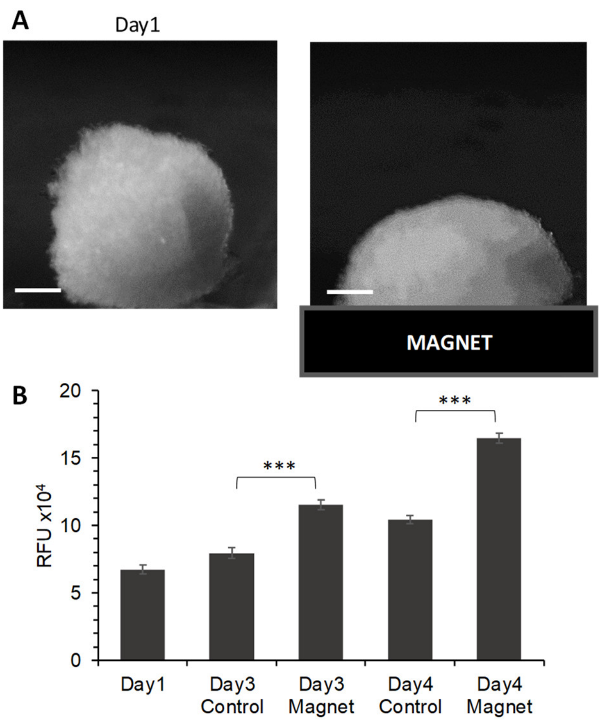

Figure S10. Formation and compression of U87 glioblastoma spheroids. Spheroids from magnetically labelled U87 cells (human glioblastoma cell line) were formed overnight with the magnetic molding procedure. The day after (day 1), they were collected. 15 were left free, and 15 were placed upon magnet. These ones were highly deformed **(A),** in the same range as for the CT26 spheroids. The spheroids metabolic activity was then measured (same protocol as for CT26 spheroids) at days 3 and 4, with the magnet left continuously over the 2 or 3 days in the magnet condition. The average fluorescence signal (n = 15) is shown in **(B)**, with a significant increase of spheroid proliferation for the magnetically compressed ones. *** $p < 0.005$.

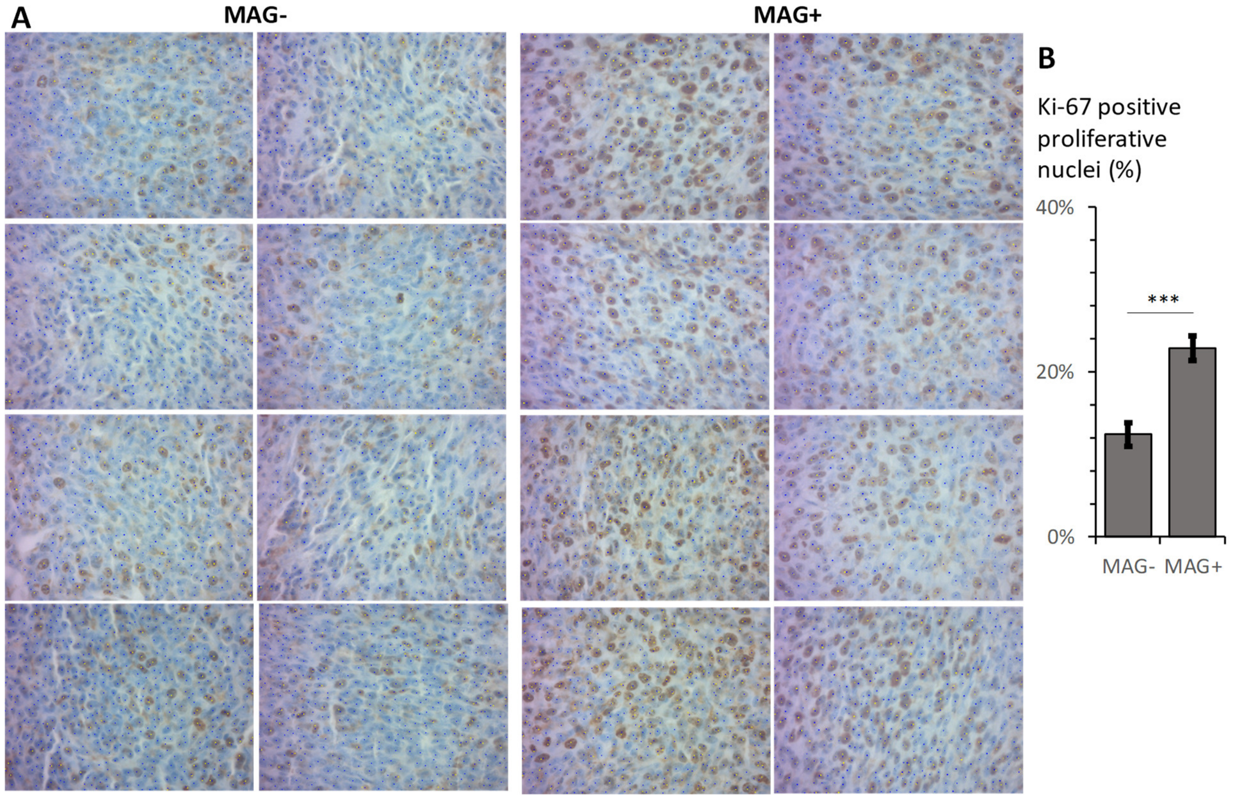

Figure S11. (**A**) Immunohistochemical staining of Ki-67 (magnification x400) on ex vivo tumor nodules collected from mice injected with MAG- control spheroids and MAG+ compressed spheroids. Blue dots and yellow dots indicate DAPI positive cells (all cells) and Ki-67 positive cells, respectively. (**B**) The ki-67 proliferation index was calculated by dividing the number of Ki67 positive cells by the total number of counted cells (400–600 cells for each image). The histogram represents the average percentage with standard error (sem) error bars ($n = 8$). ***$p < 0.005$.

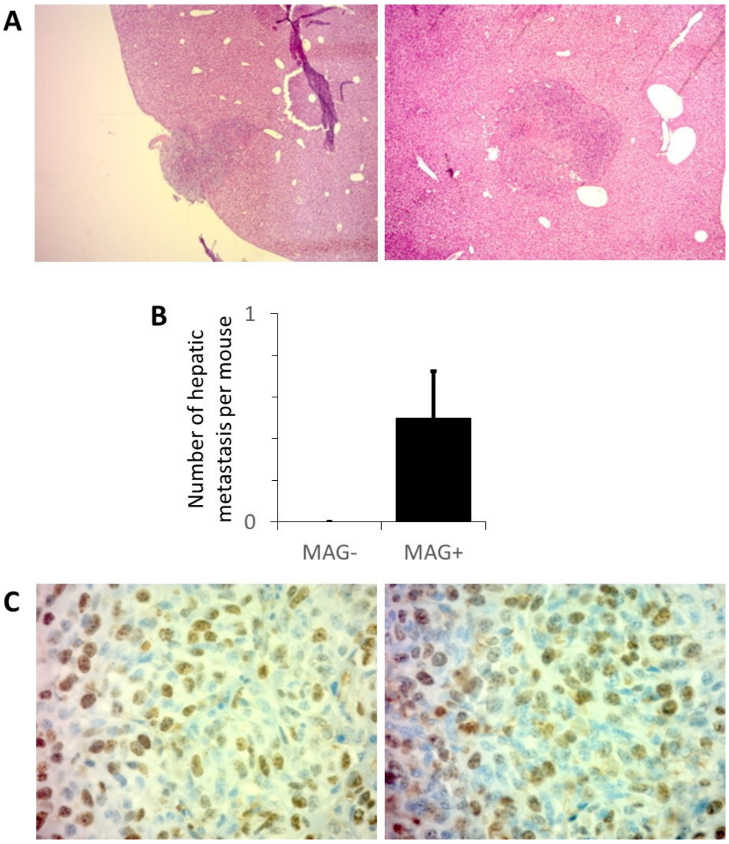

Figure S12. (**A**) Hematoxylin and eosin staining of two hepatic metastases at low magnification (×25) recovered from mice injected with compressed MAG+ spheroids. (**B**) Number of hepatic metastasis detected in mice injected with control MAG- spheroids (none) and with compressed MAG+ ones, per mouse. (**C**) Immunohistochemical staining of Ki-67 for two MAG+ spheroids derived hepatic metastases, at magnification ×400.
